# Minimally invasive skeletal sampling for STR-based human identification in degraded marine remains

**DOI:** 10.1007/s00414-026-03868-0

**Published:** 2026-06-13

**Authors:** Stefania Morelli, Giulia Cosenza, Samantha Rossini, Giulia Caccia, Debora Mazzarelli, Cristina Cattaneo, Elena Pilli

**Affiliations:** 1 IRIS -Infrastruttura per la Ricerca e l’Identificazione degli Scheletri senza nome, Dipartimento di Biologia, Università degli Studi di Firenze, Firenze, Italy; 2 LABANOF -Laboratorio di Antropologia e Odontologia Forense, Dipartimento di Scienze Biomediche per la Salute, Università degli Studi di Milano, Milano, Italy

**Keywords:** Auditory ossicles, Petrous bone micro-sampling, Short Tandem Repeats, Disaster victim Identification, Degraded skeletal remains

## Abstract

The identification of unknown skeletal remains represents a fundamental humanitarian and ethical obligation, particularly in mass fatality events and migration-related disasters, including routine forensic investigations and missing persons cases. DNA analysis is essential in these contexts, yet current sampling methods often rely on invasive procedures that compromise skeletal integrity and conflict with cultural and ethical norms. Despite major advances in forensic DNA analysis, validated strategies for minimally invasive genomic sampling in humanitarian contexts remain limited. In this study, we evaluate two minimally invasive approaches for short tandem repeat (STR) profiling -auditory ossicles and petrous bone micro-sampling- on 40 skeletal elements recovered from victims of the April 18, 2015, Mediterranean Sea shipwreck. Both strategies yield complete and high-quality (auditory ossicles) or near-complete (petrous bone micro-sampling) STR profiles while preserving cranial integrity, enabling anthropological assessment and respectful restitution. These findings demonstrate that minimally invasive genomic sampling can deliver reliable genetic data under challenging post-mortem and environmental conditions, supporting its integration into identification protocols worldwide. These findings support the implementation of minimally invasive skeletal sampling strategies in disaster victim identification workflows involving highly degraded remains.

**Supplementary Information:**

The online version contains supplementary material available at 10.1007/s00414-026-03868-0.

## Introduction

The identification of unknown human remains is a fundamental and ethical obligation, enshrined in both domestic and international law. The right to retain one’s identity after death is universally recognized and critical to mitigate psychological impact of ambiguous loss, where families remain in a state of uncertainty, not knowing whether their loved ones are dead or alive [1–4]. In mass fatality events and migration-related disasters, identification is essential not only for humanitarian reasons but also for legal and administrative purposes.

Despite international guidelines and best practices [5, 6] identifying highly degraded human remains from large-scale events -such as maritime disasters, armed conflicts, or human rights violations- remains challenging due to the complexity of the context. Factors such as the number of remains, the degree of disarticulation [7], and severe DNA degradation significantly affect the success of identification efforts [8]. This is particularly true for deeply compromised remains [9] and for maritime fatalities, where bodies submerged at sea undergo rapid post-mortem changes, including the loss of most hair and teeth and structural alterations to bone tissue.

Bones and teeth are among the most durable biological materials and often represent the only source of genetic material in these cases. However, sampling these elements is inherently invasive and compromises the integrity of the skeleton, which is crucial for anthropological assessment and respectful restitution. Preserving cranial integrity is especially important when bone lesions are present or when further analyses are required. As scientists and custodians of human dignity, we have a responsibility to minimize the impact of sampling and align identification practices with ethical and cultural expectations.

Despite major advances in forensic genetics, DNA sampling from skeletal remains still largely relies on destructive procedures that permanently alter the skull or other key skeletal elements. While these approaches maximize DNA yield, they conflict with humanitarian principles particularly in mass fatality and migration-related contexts [5, 6, 10] and limit subsequent anthropological or odontological analyses. In humanitarian operations, identification teams are increasingly required to balance analytical effectiveness with respect for human remains and the expectations of families, communities, and legal frameworks [1, 2].

Alternative skeletal elements such as the petrous bone and auditory ossicles have been previously investigated as sources of genetic material, primarily in archaeological contexts or isolated forensic casework. However, their systematic integration into standardized workflows for DVI in humanitarian settings remains limited [11–17] This gap underscores the need for validated strategies that combine technical robustness with ethical and operational sustainability.

In this study, we evaluated two minimally invasive genomic sampling approaches -auditory ossicles and petrous micro-sampling- for Short Tandem Repeats (STRs) profiling in highly degraded skeletal remains recovered from the April 18, 2015, Mediterranean shipwreck. While auditory ossicles have previously been investigated as an optimal DNA source for ancient samples [11] and for identification of highly putrefied cadavers [12], and petrous bones have recently been evaluated for forensic STR typing [13, 15, 18], their application to highly degraded skeletal remains recovered after prolonged marine submersion remains limited. Here, we specifically assessed these minimally invasive approaches in remains submerged at sea for approximately one year, providing insight into their performance under extreme environmental conditions relevant to real DVI scenarios. Beyond DNA profiling performance, we examined the implications of these methods for skeletal preservation and their integration into humanitarian identification protocols. By demonstrating that reliable genetic profiles can be obtained without highly destructive sampling, this work supports a shift toward minimally invasive, dignity-centered workflows in mass fatality management and humanitarian response.

## Materials and methods

The examinations and genetic analyses were performed within a broader governmental project dedicated to the identification of migrant victims, coordinated by the Office of the Commissioner for Missing Persons of the Italian Government and LABANOF (Laboratory of Forensic Anthropology and Odontology), University of Milan. The project involves national law enforcement authorities and multiple academic institutions. The University of Florence along with its forensic molecular anthropology lab -IRIS (Infrastructure for the Research and Identification of unknown Skeletons)- is a part of this group [19]. Out of the approximately 200 analyzed skeletal elements, 40 (20 auditory ossicles and 20 petrous bones) from as many individuals from the shipwreck that occurred in the Mediterranean Sea on April 18, 2015, were used for this study. All samples included in this study were procured from disarticulated skulls, which had not yet been associated with any individuals and had been submerged at sea for approximately one year following the shipwreck. Upon recovery, all disarticulated remains were preserved in cold storage at zero degrees Celsius, followed by a cleaning process through maceration. The petrous bones were extracted post comprehensive cleaning, while the auditory ossicles were collected as promptly as possible during or subsequent to the cleaning of the skulls. To ensure the accuracy and reliability of the molecular analysis, the recommended criteria for studies involving highly degraded DNA [20–22] were strictly adhered to, and the entire DNA analysis process was conducted in a dedicated laboratory where no other biological samples, such as blood, saliva, and semen, are processed. All testing was performed with the aim of maximizing the possibility of identification of these victims.

### Auditory ossicles sample preparation

**Fig. 1:**
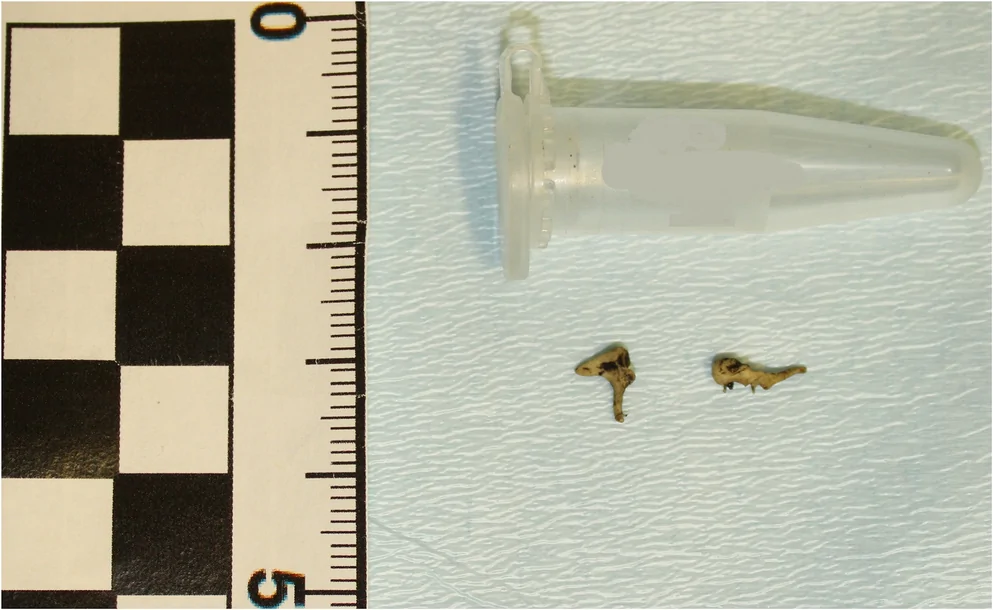
An example of an anvil on the left and a hammer on the right. The anvil was used for DNA typing

Auditory ossicles were obtained directly from the middle ear by personnel from the University of Milan using forceps. On the recommendation of personnel from the University of Florence, the anvil was preferred when available; otherwise, the hammer was chosen. Figure 1 shows a hammer and anvil from which DNA was extracted as an example.

To remove potential contaminants, the outer layer of auditory ossicles was gently cleaned with a swab moistened with DNA-free water. After cleaning, each sample was irradiated with ultraviolet light for 10 min. in a Biolink DNA Crosslinker (Biometra). The auditory ossicles were then weighed using a precision balance (Radwag, model: ps 2100/c/2), and DNA was extracted from a range of 11–29 mg of bone.

### Petrous bones sample preparation

**Fig. 2:**
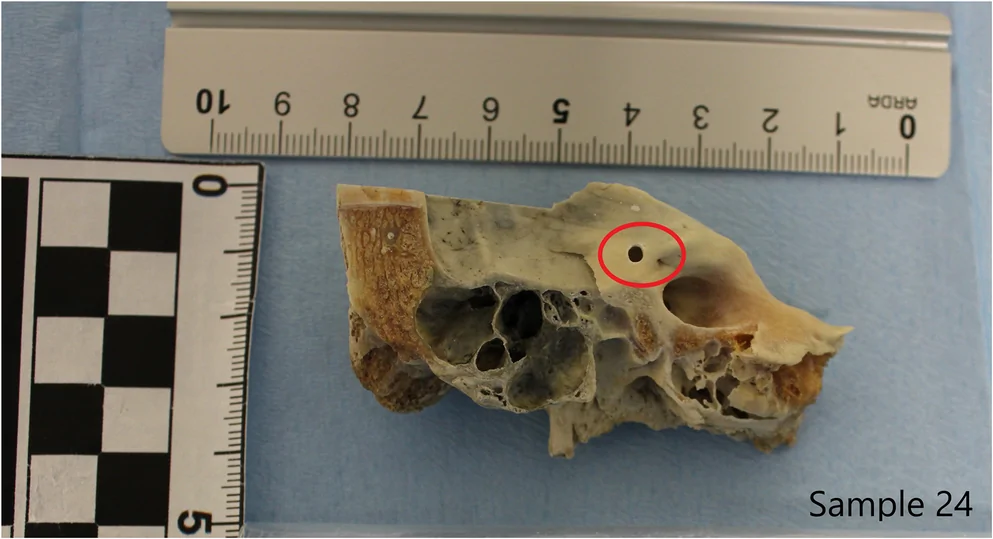
The micro-hole from which the bone powder was collected from DNA typing is indicated by the red arrow

Sampling of the petrous bone was conducted by personnel from the University of Milan using a Stryker saw. This process completely removed the temporal bone region, including the mastoid process, external auditory meatus, and petrous bone. At the IRIS lab, to eliminate potential contaminants, the outer layer of each petrous bone was mechanically removed using a low-speed rotary sanding drill (Dremel^®^ 300 series). Subsequently, each sample was irradiated with UV light for 45 min in a Biolink DNA Crosslinker (Biometra). As proposed by Sirak et al. [23]., approximately 50 mg of bone powder was sampled by micro-drilling the petrous bones in order to keep the petrous bone as intact as possible. Figure 2 shows petrous bone microsampling as an example.

After micro-sampling, the petrous bones underwent axial computed tomography (CT) analysis to assess the effectiveness of micro-sampling techniques in reaching the cochlea, which is a crucial site for DNA analysis [14]. This evaluation was conducted at the Galeazzi Orthopedic Institute in Milan, employing the Newtom 7G. DICOM-formatted files were analyzed using Slicer 4.13 software [24].

### DNA extraction and quantification

DNA was extracted from all samples following a published silica-based protocol [25] and eluted in 100 µl of TE buffer (10 mM Tris, 1 mM EDTA) with TET buffer (0.05% Tween-20). The extracts were then quantified using the Agilent 2100 Bioanalyzer System and the High Sensitivity DNA kit (Agilent Technologies). Although the TapeStation/Bioanalyzer platform is not human-specific and does not replace quantitative PCR methods routinely used in forensic casework, its use in this study was limited to assessing DNA fragmentation patterns and overall template availability prior to STR amplification. Therefore, quantification values were interpreted cautiously.

### Short tandem repeat (STR) typing

STR typing of autosomal DNA was performed using the PowerPlex^®^ Fusion 6 C System kit (Promega Corporation), capable of simultaneously amplifying 27 loci, meeting both CODIS and ESS recommendations. In addition to the samples, positive (2800 M Control DNA, Promega) and negative PCR and extraction controls were also amplified. Reaction was carried out on a SureCycler 8800 Thermal Cycler (Agilent Technologies) according to the manufacturer’s instructions. PCR products were separated via capillary electrophoresis on the SeqStudio Genetic Analyzer (Applied Biosystems) Cartridge v2, a 4-capillary electrophoresis system utilizing the universal POP-1 polymer. Genetic profiles were determined using the public domain, free, and open-source computer software Osiris, validated for clinical, forensic, and research applications [26–28]. The analytical threshold was set at 150 relative fluorescence units (RFU) for all auditory ossicles and for 9 out of 20 petrous bone micro-sampling. Profile completeness was defined based on successful amplification of all loci above the analytical threshold and consistency across replicate analyses, where performed, to support profile reliability. For the remaining 11 petrous bone samples, the threshold was set at 50 RFU, in accordance with forensic practice for highly degraded or low-template DNA samples. Lower thresholds may increase stochastic effects such as allelic dropout and drop-in in low-template DNA samples. However, replicate PCR amplifications were performed for selected samples, particularly for the 11 petrous bone samples requiring increased interpretative caution following the application of a reduced analytical threshold. Replicates were carried out from the same DNA extract, and consensus profiles were generated by considering only alleles consistently observed across independent amplifications, in accordance with established forensic DNA interpretation principles [29].

Statistical comparisons of RFU values per sample between auditory ossicles and petrous bone samples were performed using the non-parametric Mann–Whitney U test implemented in R. Effect size (r) was calculated to assess the magnitude of differences.

## Results and discussion

DNA was successfully extracted from all skeletal elements, and quality and quantity were initially assessed using the Agilent 2100 Bioanalyzer System. Although this instrument is not the standard for forensic quantification, its use provided valuable insights into DNA degradation patterns, informing subsequent STR analysis.

### Auditory ossicles

Quantification values, assessed within the size range (70–500 bp) of amplified fragments, varied from 84.03 pg/µl to 876.32 pg/µl. Each sample produced a detectable signal within this range. In particular, the results highlighted that 15% of the samples showed quantification values below 200 pg/µl, with just one sample below 100 pg/µl. 40% of the samples had concentration values of approximately 200/400 pg/µl and the remaining 35% showed concentration values above 400 pg/µl with two samples (10% of the total) exceeding 690 pg/µl. Fig. S1 shows an example of quantification profiles of four auditory ossicles.

**Fig. 3:**
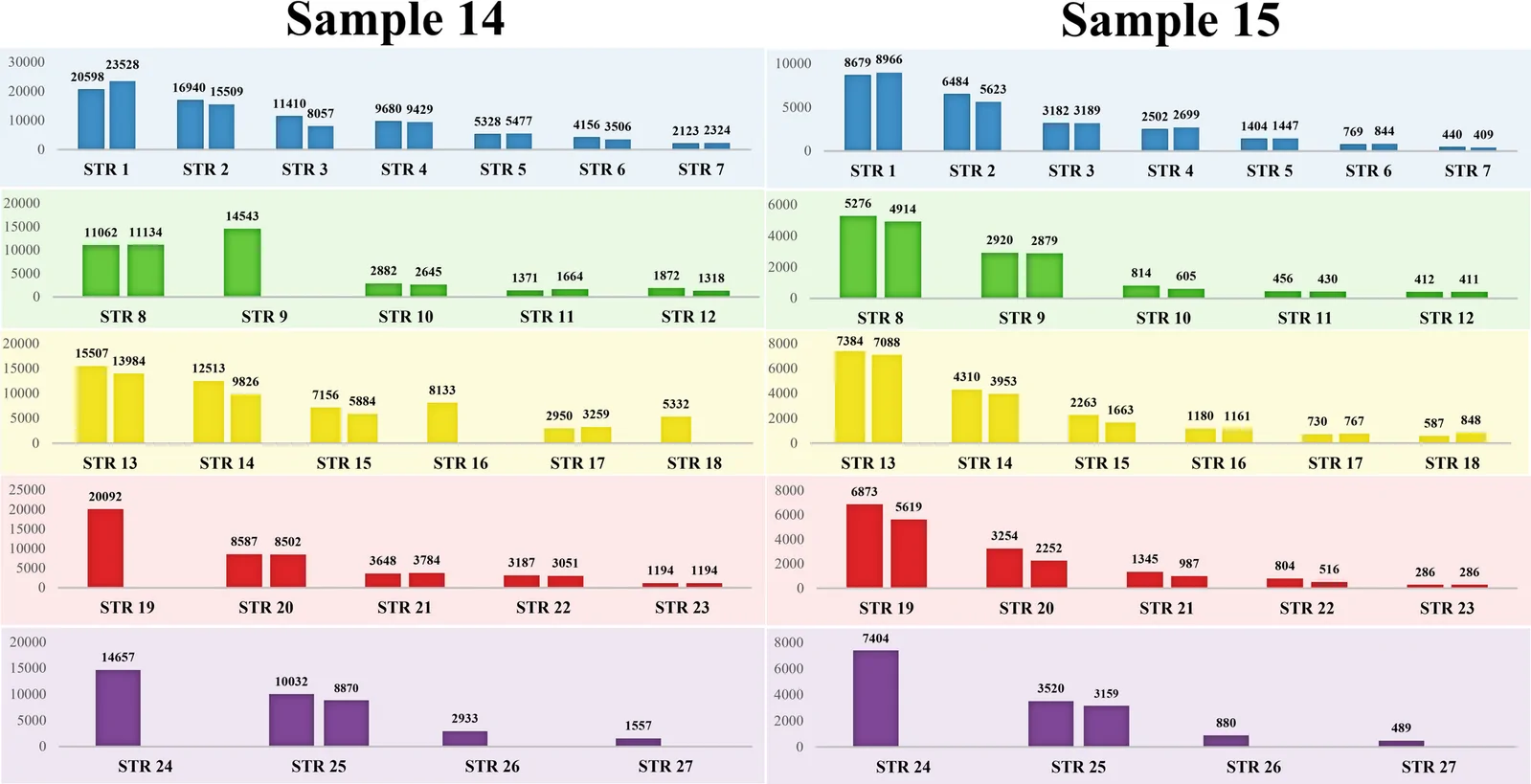
The auditory ossicles reproduced STR profiles of samples 14 and 15, representing respectively the “best” and “worst” profile obtained among the four auditory ossicles quantification (Fig. S1)

In addition to quantification information, electropherograms provided an assessment of the quality and size of the DNA extracts, showing a fairly homogeneous fragmentation profile over the range considered, with an increase in quantity around between 70 and 250 bp. Despite the DNA degradation and the presence of non-human DNA (Agilent analysis is not specific for quantifying human DNA), the results seemed to indicate that there was sufficient DNA to proceed with DNA typing. Therefore, STR typing was carried out on all extracts. According to quantification results, a complete and high-quality profile was obtained for all 20 analyzed auditory ossicles, with all 27 loci successfully typed. Figure 3 shows the reproduction of two STR profiles from auditory ossicles 14 and 15, for privacy reasons.

**Fig. 4:**
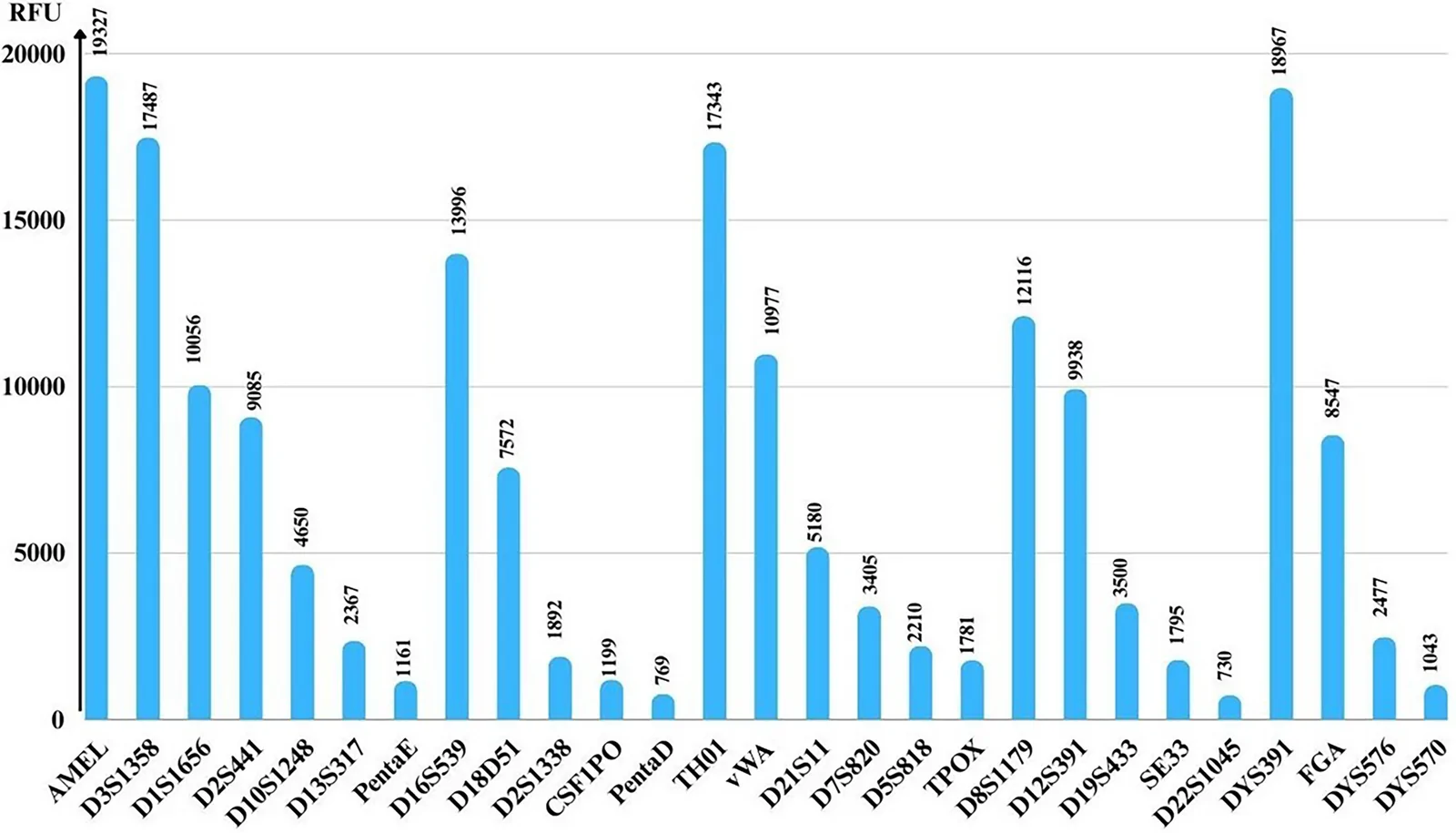
Average RFU values calculated for each locus in the auditory ossicles’ profiles. Loci were arranged according to the fluorescent dye channels of the Powerplex^®^ Fusion 6 C System kit. The decrease in RFU values with increasing amplicon size reflects the typical “decay curve” observed in degraded DNA samples, where shorter fragments amplify more efficiently than longer ones

As expected when amplifying degraded DNA samples, signal intensity decays as the size of the PCR product increases, and a “decay curve” is observed in which the peak height is inversely proportional to the amplicon length [30–33] (Fig. 4).

As can be observed in Fig. 4, PCR products of approximately 100 bp (for example, D31358, D16S539, TH01, D8S1179, and DYS391) exhibited a higher signal compared to PCR products of approximately 400 bp (such as Penta E, Penta D, TPOX, D22S1045, and DYS570). This is typically because a greater number of DNA molecules remain intact within a smaller size range as opposed to a larger one. Nevertheless, even for the largest PCR products, notably high RFU values were obtained, with mean values spanning from 730 for the D22S1045 locus to 1781 RFU for TPOX. In particular, focusing on the evaluation of the largest individual PCR products (Table S1), it can be seen that these in all samples greatly exceed the analytical threshold of 150 RFUs. In addition, 55%, 25%, 60%, 30%, and 40% of the samples showed RFU values well above 1000 for Penta E, Penta D, TPOX, D22S1045, and DYS570, respectively.

These findings, supported by average RFU values ranging from 2585 to 14,255 RFUs, indicate that auditory ossicles maintain exceptional DNA preservation despite prolonged exposure to harsh environmental conditions, such as over one year submerged at sea. These values are significantly higher than those reported by Schwart et al. [12], who observed similar values only in cases without sign of putrefaction, whereas ossicles in our study performed well even under extreme degradation. This confirms that STR typing can be successfully applied to fully skeletonized remains recovered long after death, producing complete profiles of excellent quality. The robustness observed is likely linked to the extraction protocol optimized for highly degraded bone samples. Importantly, these results highlight that auditory ossicles are a largely underutilized resource for identification in humanitarian contexts. Their ability to yield complete, high-quality STR profiles while preserving cranial integrity supports their systematic inclusion in DVI workflows. By enabling genetic analysis without destructive sampling, ossicles safeguard anthropological information and align identification practice with ethical and cultural expectations, reinforcing dignity in mass fatality management [9, 10, 14].

### Petrous bones micro-sampling

DNA concentrations, assessed within the size range (70–500 bp) of amplified fragments, ranged from 34.16 pg/µl to 779.01 pg/µl. Each sample produced a detectable signal within this range, although in some samples (samples 21 and 24 Fig. S2) the low amount of DNA made signal detection difficult. 45% of the samples (9 out of 20) registered concentrations over 300 pg/µl, including one sample with a quantification value significantly surpassing 700 pg/µl. Three samples presented quantification values under 100 pg/µl and another 40% had values ranging from 100 to 300 pg/µl.

Figure S2 shows an example of quantification of four micro-samples from petrous bones.

As can be observed in Fig. S2, a smear analysis was performed, and the estimated concentration values for the four samples were just over 30 pg/µl for sample 21, 150 pg/µl for sample 24, 300 pg/µl for sample 31, and approximately 200 pg/µl for sample 35. Additionally, the electropherograms were evaluated to assess the quality and size of the DNA extracts, revealing similar fragmentation profiles across the analyzed range. No increase in the quantification values was observed between 70 and 250 bp, with similar results along the entire range, in contrast to auditory ossicles, which showed a peak between 70 and 250 bp compared to 250–500 bp. A common feature observed in all petrous samples, regardless of DNA amount, was a distribution curve rising toward fragment lengths (sample 31 in Fig. S2) above 1000 bp, indicating possible exogenous DNA contamination.

**Fig. 5:**
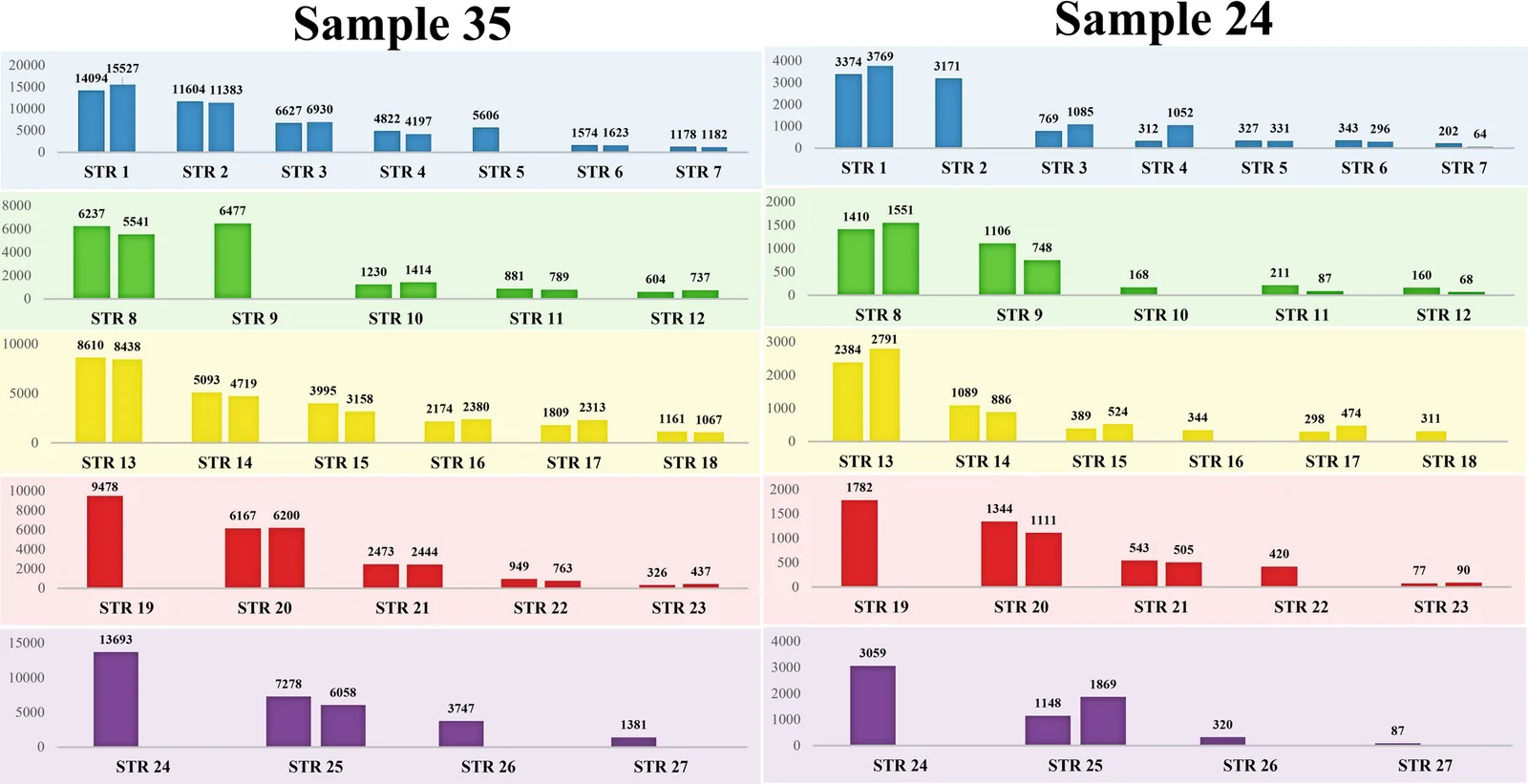
The reproduced STR profiles from micro-sampling of petrous bone 35 and 24, representing respectively the “best” and “worst” complete profile obtained among the four micro-sampling quantification (Fig. S2)

Following quantification, STR typing was performed on all extracts. Complete STR profiles were successfully generated from 85% (17 out of 20) of the samples analyzed. To obtain reliable STR profiles, eleven of these samples required replication and consensus profiles were generated. Partial profiles with 18, 19, and 21 loci typed were obtained from the remaining 15%. Figure 5 shows examples of two STR profiles from petrous bones 24 and 35.

In summary, all samples attained at least 18 STR loci, exceeding CODIS guidelines (13 loci) [34] and Italian standards (10 loci) [35].

Although high DNA quantification values were observed, this did not consistently yield complete, high-quality STR profiles. A limitation of the quantification approach used in this study is that the Agilent Bioanalyzer system does not distinguish between endogenous human DNA and exogenous DNA, including environmental or microbial contamination. This is particularly relevant for petrous bone samples, where electropherograms suggested the presence of high-molecular-weight DNA fragments, likely reflecting non-human DNA. As a result, quantification values may overestimate the amount of amplifiable human DNA, which could explain the discrepancy observed between DNA quantity and STR profile quality in some samples.

**Fig. 6:**
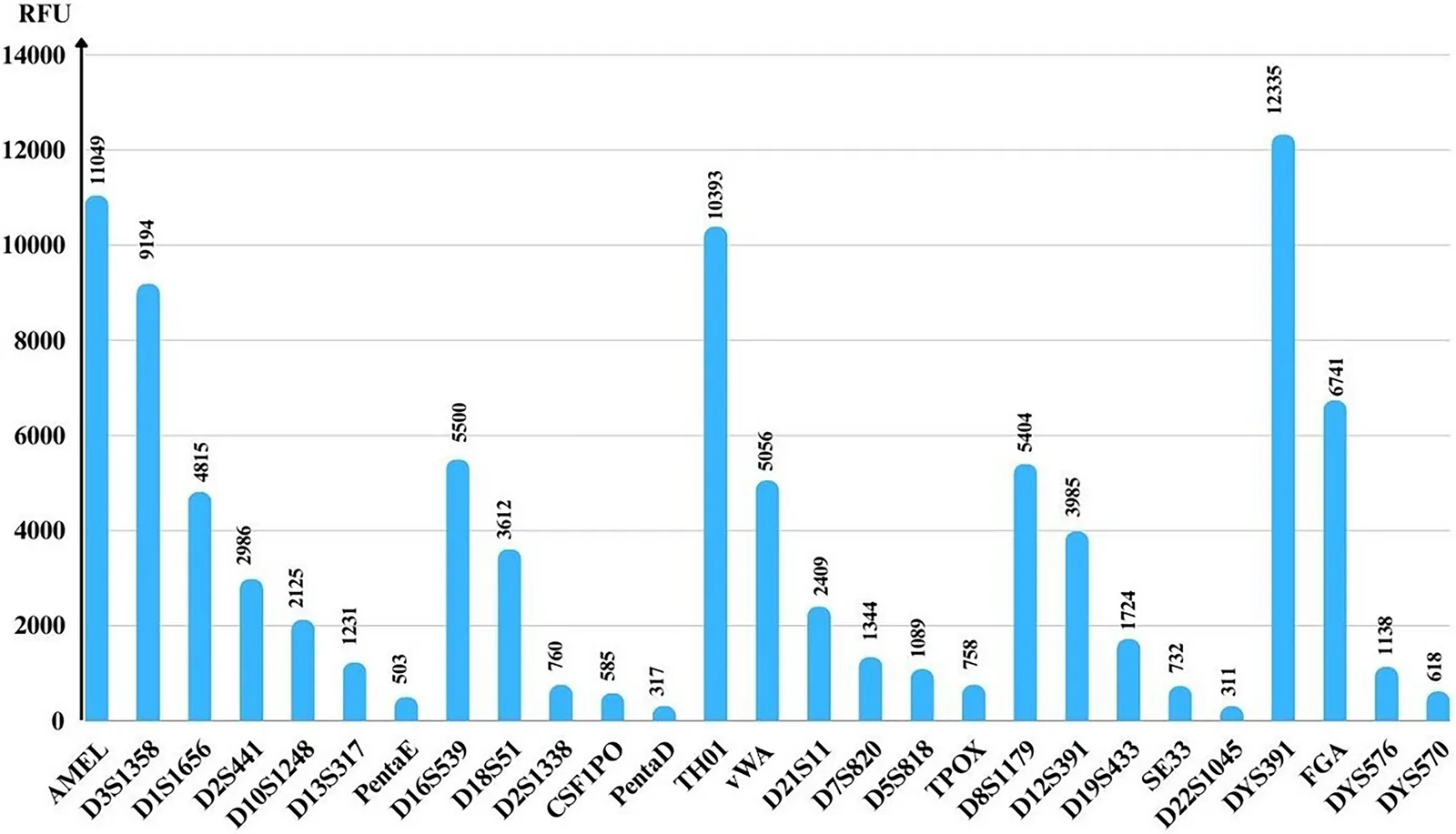
Average RFU values calculated for each locus (considering only detected alleles) in the petrous bone profiles. Loci were arranged according to the fluorescent dye channels of the Powerplex^®^ Fusion 6 C System kit. A more pronounced decay curve is observed compared to auditory ossicles, indicating greater DNA degradation, with reduced amplification efficiency of longer STR loci

In some cases, replication was necessary, and the analytical threshold was set at 50 RFU, as for complex degraded traces. The use of a reduced analytical threshold increases sensitivity but may also increase stochastic effects; however, the use of replicate analyses and consensus profiling mitigates this risk and is consistent with forensic practice. Qualitative evaluation of electropherograms explains these results: graphs (Fig. S2) show low amounts of short DNA fragments, that is, those of the size expected for STR typing, but a notable presence of large fragments (evidenced by the hump present around 2000 bp), likely indicating contamination or non-human DNA. Signal strength decreased with increasing PCR product size, resulting in a more pronounced “decay curve” compared to auditory ossicles. This curve highlights that the peak height is inversely proportional to the length of the amplicon, as observed in Fig. 6.

**Fig. 7:**
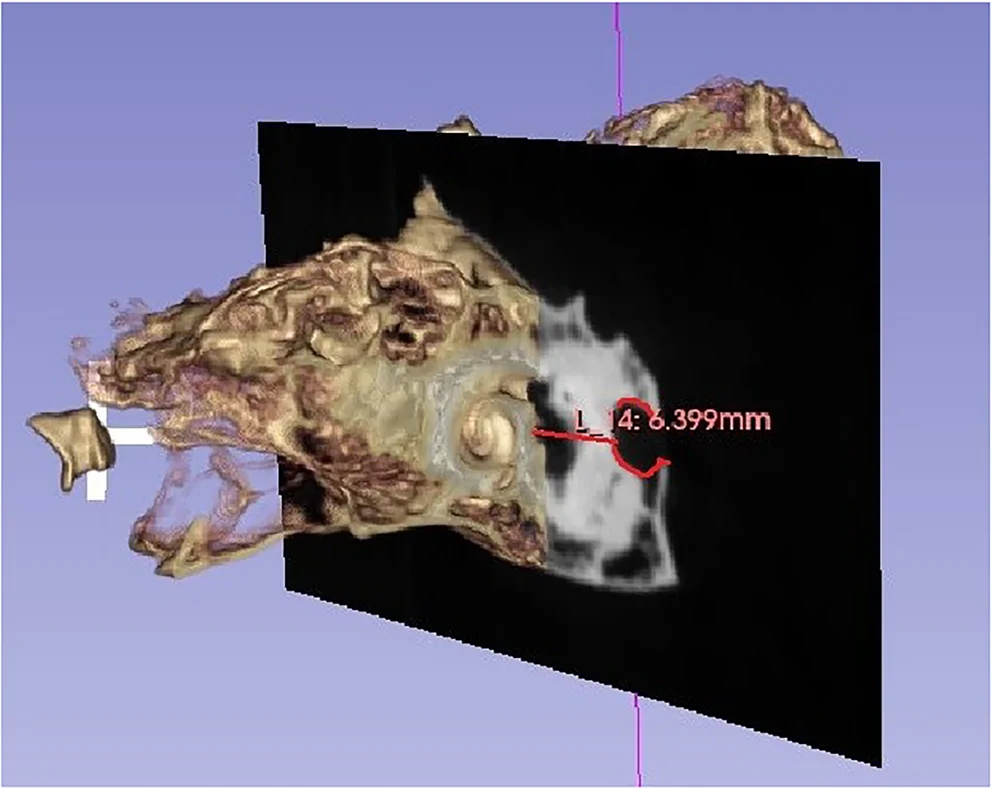
Computed tomography of petrous bone micro-sampling 24. The figure, with the sampling point circled in red, allows the distance (indicated by red line) between the sampling point and the cochlea to be assessed

Average RFU values ranged from 268 to 10,858 per profile, confirming that petrous bone DNA is degraded but sufficient for complete or near-complete STR profiles. Anatomical variability also influenced results. Previous studies [13, 36] showed that the inner-ear portion (zone C) provides optimal endogenous DNA. Our micro-sampling strategy [23] preserved cranial integrity by CT scans confirmed that sampling never reached zone C, remaining about 6 mm away (Fig. 7). These observations highlight that petrous bone micro-sampling can preserve cranial integrity but requires careful planning and anatomical assessment. Pre-sampling CT imaging is recommended to optimize DNA yield while maintaining minimally invasive principles. Even without reaching the cochlear region, our results demonstrate that micro-sampling provides genetic profiles suitable for identification, supporting its use when less invasive alternatives are required.

However, despite this recommendation to optimize DNA yield, our analysis demonstrated that STR typing can still be successfully performed using micro-sampling without altering cranial integrity, supporting its application in humanitarian identification workflows.

### Pros and cons in the choice of sampling

A key limitation of this study is the lack of paired sampling, as auditory ossicles and petrous bones were not collected from the same individuals. This introduces inter-individual variability and limits direct comparability between the two sampling strategies. Therefore, the observed differences in DNA yield and STR performance should be interpreted with caution and not as definitive evidence of the superiority of one method over the other. The primary aim of this study was to evaluate the feasibility of minimally invasive sampling approaches in a real DVI context, rather than to perform a direct comparative assessment under controlled conditions.

In this section, we summarize the main advantages and limitations of auditory ossicles and petrous bone micro-sampling for humanitarian identification in mass fatality contexts. This is not intended as a point-by-point comparison, which would require sampling both skeletal elements from the same individual.

1. *Sample collection and preparation*: Both approaches can be considered minimally invasive, although auditory ossicles involve virtually no structural alteration compared to petrous bone micro-sampling. However, their small size makes them prone to being overlooked or lost during recovery. Ossicles are also simpler to clean and do not require pulverization, eliminating a step in the laboratory workflow and reducing contamination risk. This translates into faster processing and improves operational efficiency.
2. *Quantification results*: DNA quantity and quality varied widely, from < 100 pg/µl to > 800 pg/µl for auditory ossicles and up to approximately 700 pg/µl for petrous bone micro-sampling. Generally, ossicles tended to yield higher DNA amounts in this dataset in the 70–500 bp range, which are compatible with STR loci and natural degradation patterns. In contrast, petrous bone samples displayed a more uniform distribution across the analyzed range, without a clear increase in the short-fragment region, and often showed a hump around 2000 bp, likely due to exogenous or non-human DNA introduced during sampling. This explains why high quantification values in petrous bones did not always correspond to complete STR profiles.
  In general, the results obtained from quantification suggested a higher amount of DNA in the auditory ossicles compared to the micro-sampling of petrous bones. These results were associated with complete and generally higher quality STR profiles in the auditory ossicles within this dataset, although direct comparison is limited by the study design. Therefore, although quantification is non-specific for human DNA, it can still provide an indication of the quantity and preservation state for subsequent multiplex PCR analysis.
3. *STR typing*: Both methods exhibited the expected decay curve for degraded DNA, with peak heights inversely related to amplicon size (Figs. 4 and 6). Comparing these curves (Figs. 4 and 6), auditory ossicles appeared to yield more DNA suitable for STR typing than petrous bones micro-sampling under the conditions of this study when the same PCR input was used. This was evident from consistently higher peak heights across all markers. Moreover, DNA from ossicles appeared less degraded, as indicated by lower proportional decreases within each fluorophore compared to petrous bone samples, which showed more extensive degradation. Consequently, complete STR profiles of excellent quality were obtained from all ossicles without PCR replication, whereas petrous bone micro-sampling produced complete profiles in 17 of 20 cases, with 11 requiring replication. A statistical comparison of RFU values per sample showed significantly higher values in auditory ossicles (median = 6144) compared to petrous bone samples (median = 1879; Mann–Whitney U test, W = 308, *p* = 0.0029), with a moderate effect size (*r* = 0.47). These results support the observed differences in amplification performance, although they should be interpreted with caution due to the lack of paired sampling. These profiles enabled identification of 19 individuals recovered from the Mediterranean Sea through standard DVI comparison procedures. Overall, these findings support a flexible, context-driven approach to skeletal sampling, prioritizing minimally invasive strategies to maximize identification success while preserving human remains in humanitarian operations.

### Operational considerations for minimally invasive sampling in DVI contexts

The results of this study support the structured integration of minimally invasive skeletal sampling strategies into disaster victim identification (DVI) workflows involving highly degraded remains. Based on the comparative performance of auditory ossicles and petrous bone micro-sampling, several operational considerations emerge.

1. *Preservation of anatomical integrity.* Whenever feasible, sampling strategies should prioritize skeletal preservation, particularly of cranial structures that are critical for anthropological, odontological, and pathological assessment. Auditory ossicles can be removed with negligible structural alteration and without compromising subsequent forensic examination. Petrous bone micro-sampling, when performed using controlled micro-drilling techniques and anatomical guidance, can preserve cranial integrity while still providing sufficient DNA for STR typing.
2. *Balancing invasiveness and diagnostic yield.* Sampling decisions should consider the expected DNA yield in relation to the degree of skeletal disruption. In the present study, auditory ossicles consistently yielded complete STR profiles without the need for replication, supporting their use as a first-line option when available. Petrous bone micro-sampling produced complete or near-complete profiles in the majority of cases and represents a valid alternative when ossicles are absent or inaccessible. The moderate effect size suggests that, while auditory ossicles generally provide higher RFU values, petrous bone samples may still yield comparable results in selected cases. This supports a flexible, context-dependent sampling strategy rather than a strict preference for a single method.
3. *Replication and analytical thresholds in degraded samples.* In highly degraded contexts, sensitivity must be balanced with reliability. The requirement for allele replication and the adjustment of analytical thresholds in selected cases reflect established forensic practice for degraded skeletal material. Conservative interpretation criteria remain essential to prevent erroneous inclusions or exclusions in DVI scenarios.
4. *Integration within multidisciplinary identification procedures.* Minimally invasive genomic sampling should complement, rather than replace, anthropological, odontological, and contextual analyses. The approaches evaluated here are compatible with established DVI protocols and may enhance identification efficiency while maintaining structural preservation of remains.

Together, these considerations provide a practical framework for implementing minimally invasive skeletal sampling in forensic casework and mass fatality investigations involving degraded maritime remains.

## Conclusions

To develop innovative approaches for skeletal sampling that optimize DNA typing while preserving the structural integrity of remains, this study evaluated two promising strategies: auditory ossicles and petrous bone micro-sampling. Our findings demonstrated that auditory ossicles are highly effective for obtaining high-quality DNA, simplifying collection and minimizing disruptions to the skull. When ossicles are unavailable, petrous bone micro-sampling offers a viable alternative, producing complete or near-complete STR profiles in most cases while maintaining cranial integrity. This technique should be performed with the bone in its anatomical position by trained specialists to ensure minimal invasiveness and structural preservation.

Beyond technical performance, these results have broader implications for forensic and humanitarian operations. Identification efforts -whether in mass fatality events, migration-related disasters, or routine missing persons cases- must balance scientific effectiveness with ethical responsibility, while respecting dignity and cultural values. By showing that robust genetic profiles can be obtained without highly destructive sampling, this work supports a paradigm shift toward more sustainable, ethically grounded workflows. Integrating these approaches into identification protocols will strengthen international standards and provide families with answers while honoring the integrity of human remains.

## Supplementary Information

Below is the link to the electronic supplementary material.

Supplementary Material 1 (DOCX 267 KB)
