# Supplementary material for: Minimally invasive skeletal sampling for STR-based human identification in degraded marine remains
Source: Int J Legal Med. 2026 Jun 13;140(5):2697–707. doi: 10.1007/s00414-026-03868-0 (PMC13499827; doi:10.1007/s00414-026-03868-0)
Supplement: Supplementary file 1 [file 414_2026_3868_MOESM1_ESM.docx]

**Minimally invasive skeletal sampling for STR-based human identification in degraded marine remains**

*Stefania Morelli^1^, Giulia Cosenza^1^, Samantha Rossini^1^, Giulia Caccia^2^, Debora Mazzarelli^2^, Cristina Cattaneo^2^, Elena Pilli^1^.*

^1^IRIS -Infrastruttura per la Ricerca e l’identificazione degli Scheletri senza nome- Dipartimento di Biologia, Università degli Studi di Firenze, Firenze, Italy.

^2^LABANOF -Laboratorio Di Antropologia E Odontologia Forense-, Dipartimento di Scienze Biomediche per la Salute, Università degli Studi di Milano, Milano, Italy.

Corresponding author: Elena Pilli IRIS (Infrastruttura per la Ricerca e l’identificazione degli Scheletri senza nome) Department of Biology, University of Florence. Via del Proconsolo 12, 50122 Firenze, Italy. Email: [elena.pilli@unifi.it](mailto:elena.pilli@unifi.it)

**Fig. S1** DNA smear analysis for four auditory ossicles. On the Y axis is the DNA concentration expressed in fluorescence units (FU) and on the X axis is the fragment length in base pairs. The % of total, average size, concentration, and molarity are automatically determined and displayed in the region table.

**Fig. S2** Quantitative electrophoresis results for four petrous bones. On the Y axis is the DNA concentration expressed in fluorescence units (FU) and on the X axis is the fragment length in base pairs. The % of total, average size, concentration, and molarity are automatically determined and displayed in the region table.

|  | **PentaE** | **PentaD** | **TPOX** | **D22S1045** | **DYS570** |
| --- | --- | --- | --- | --- | --- |
| **average RFU** | **n° of samples** | **n° of samples** | **n° of samples** | **n° of samples** | **n° of samples** |
| **for *locus*** |  |  |  |  |  |
| **100-300** | 0 | 3 | 0 | 5 | 1 |
| **300-500** | 3 | 6 | 0 | 5 | 3 |
| **500-700** | 3 | 3 | 2 | 1 | 4 |
| **700-1000** | 3 | 3 | 6 | 3 | 4 |
| **1000-2000** | 8 | 4 | 5 | 6 | 8 |
| **>2000** | 3 | 1 | 7 | 0 | 0 |

**Table S1** Distribution of average RFU values for single allele for PentaE, PentaD, TPOX, D22S1045, and DYS570 loci
